# Epidemiology and treatment of pediatric tibial fractures in Sweden: a nationwide population-based study on 5828 fractures from the Swedish Fracture Register

**DOI:** 10.1007/s00068-022-02157-w

**Published:** 2022-11-05

**Authors:** Matilda Gothefors, Olof Wolf, Yasmin D. Hailer

**Affiliations:** Section of Orthopedics, Department of Surgical Sciences, Uppsala University, Uppsala, Sweden

**Keywords:** Epidemiology, Swedish Fracture Register, Tibia fracture, Pediatric, Seasonal variation, Treatment

## Abstract

**Purpose:**

Pediatric tibial fractures have been described internationally as mainly caused by fall during leisure activities and organized sports and showing a higher incidence in boys. Still, most studies are single center studies or have a small sample size. This study aimed to analyze sex and age distribution, seasonal variation, injury mechanisms and treatment of pediatric tibial fractures based on the nationwide Swedish Fracture Register (SFR).

**Methods:**

All tibial fractures in patients < 16 years at injury and registered in 2015–2019 were extracted from the SFR. We analyzed patient characteristics such as sex and age, injury mechanism, fracture location and treatment.

**Results:**

The study cohort consisted of 5828 pediatric tibial fractures in 5719 patients. Median age of the patients was 7 years and 58% were boys. Shaft fractures were most common, followed by the distal and proximal tibia. The lowest incidence was observed during autumn. The most common cause of injury was fall mostly involving winter sports, stumbles and fall from play equipment. Play/free time and sports were the most common activities, common places of injury were sports facility and home. 1% were open fractures. 78% were treated non-surgically. Screw fixation was performed in 52% of surgically treated fractures, predominantly in the distal segment.

**Conclusion:**

Injury mechanism differs between age groups; play/free time injuries are common in younger children compared with sport activities in older children. Most patients are treated non-surgically. Open fractures are rare. Information on injury patterns is useful working preventively, for example safety work in playgrounds.

## Introduction

Internationally, tibial fractures account for about 15% of the long-bone fractures in children and adolescents [1]. The most common injury mechanisms are falls [2, 3] during sports and leisure activities [2, 4–6] and occur in summer [7]. Most pediatric tibial fractures are shaft fractures [8]. A higher overall fracture incidence is described in boys [2–11], with a peak at age 14 in boys and 11 in girls [11]. Most pediatric tibial fractures can be treated non-surgically [1, 9, 12–15].

International studies are often single center studies with smaller study groups or focus on specific types of tibia fractures showing variations in the overall fracture risk and fractures of the tibia [16]. The existent epidemiological data may not be applicable for the Swedish pediatric population as environment and injury mechanisms and treatment regimens may vary internationally. Since 2015, the Swedish Fracture Register (SFR) includes data of fractures in children. There are a few published studies on Swedish pediatric fractures in all locations [10, 17, 18], but none of the published studies is specifically regarding tibia fractures, and none is based on nationwide data from the Swedish pediatric population.

This study aimed to answer the following research questions:

- What are common population characteristics, fracture locations and injury mechanisms for pediatric tibial fractures in Sweden?
- Is there a seasonal variation in the incidence of tibial fractures in Sweden?
- What is the most common treatment strategy in Sweden?
- Which type of treating physician mainly treats pediatric tibial fractures when surgical treatment is chosen?

## Materials and methods

### Data collection

#### Study design and setting

This is an observational study on the epidemiology of pediatric tibial fractures in Sweden. The study is based on data extracted from the Swedish Fracture Register (SFR). The SFR is a nationwide quality register established in 2011 where treating physicians register all fractures and accompanied information such as injury mechanism, patient characteristics (age and sex) and performed treatment. Patients with a fracture are individually registered through the SFR’s online platform after secure login [19]. Together with the patient’s individual personal identity number, detailed information on injury mechanism and low or high energy injury as well as pathological or stress fracture as the cause of injury is entered. Thereafter, the fracture or fractures are added and classified using mainly the AO/OTA 2007 classification. The pediatric classification tool for long bones was added in 2015. Treatment is registered as surgical and non-surgical. The surgical methods are further subdivided as described under outcome variables below. Reoperations can later be added should they occur. Due to the stepwise introduction of the SFR, both coverage and completeness have increased over the years. Coverage reached 100% in early 2021 with all orthopedic departments activated in SFR.

#### Patient selection

All patients < 16 years at injury with a registered tibial fracture (ICD code S82.10/11/20/21/30/31) sustained between January 1 2015 and December 31 2019, including refractures. Refractures are defined as fracture with the same ICD-code and fracture site that occurred at least 7 days after the primary registered fracture.

#### Outcome variables

This study analyzes sex, age, fracture localization (proximal, shaft and distal tibia fractures), injury mechanism (cause, activity, place), injury type (high or low energy trauma), open or closed fracture, injury season, performed primary treatment and level of treating physician.

The patients were divided in age groups, 0–3, 4–6, 7–9, 10–12 and 13–15 years, which has been used in previous epidemiologic fracture studies [20].

In the SFR, fractures are classified both according to the AO-classification system and according to fracture localization/skeletal segment; proximal, diaphyseal (shaft) or distal. We have chosen to report our results divided by skeletal segment.

Injury cause variables were divided in fall, transport accidents, exposure to living forces, exposures to mechanical forces and other. Subgroup variables with a frequency < 100 fractures were combined. The combined group “other fall” included fall from bed, tree, chair, building, cliff, ladder, wheelchair, scaffolding, diving, jumping or falling from height and fall when carried or supported. Under transport accidents, “other transport accident” included pedestrian, horse riding, tricycle, car, watercraft, funicular, bus, “other vehicle” and unspecified. Under living forces, “other living forces” were a combination of being hit or bitten by another person, squeezed by crowd, bitten by animal and other living forces. Under mechanical forces, “other mechanical forces” were a combination of being squeezed between objects, exposed to thrown or falling object, other objects and other machine. Lastly, “other” causes include stress fracture, spontaneous fracture, abuse, intentional self-destructive action, exposure to natural forces and other factors, primary tumor and other accident.

Injury activity variables were divided in play/free time, sports and other specified. The activity group “other specified” was variables with low frequencies and include work, other and rest/hygiene/meal.

Place variables were divided in sports facility, home, school/hospital/public premises and other specified. “Other specified” places include street, road, institutional housing, store area/service area, industrial area/construction and agricultural area.

Regarding seasonal variation, months were divided in seasons; winter (Dec–Feb), spring (Mar–May), summer (Jun–Aug) and fall (Sep–Nov).

Treatment is registered as surgical or non-surgical where surgical treatment includes all treatments under anesthesia. The surgical treatment is further specified in screw fixation, K-wire, intramedullary nail, external fixation, plate fixation, fixation with bio implant, extraction of implant, other fracture fixation/combined method and other surgery. Type of surgery method is also divided into primary, secondary, tertiary treatment.

#### Statistics

Continuous data were described using means, medians, ranges, and standard deviations, as appropriate, and differences between observed and expected counts of categorical data were investigated by the Chi-square test and Bonferroni post-hoc test. Continuous variables were analyzed using the Mann–Whitney *U* tests as most of these were nonparametric. The level of significance was set at *p* < 0.05 in all analyses. Multivariable logistic regression models were used to calculate the risks for fracture location and surgery as the dependent variable in relation to age, sex and season of fracture occurrence and reported as Odds ratio (OR) with 95% confident interval (CI). The statistical analyses were calculated in R software (R version 4.0.5 (2021–03-31). The tables were created with IBM SPSS statistics (version 28.0.1.0).

#### Ethics

Ethical approval from the Swedish Ethical Review Authority was obtained before the start of this study (D-nr 2019-04282).

## Results

### Population characteristics

**Fig. 1:**
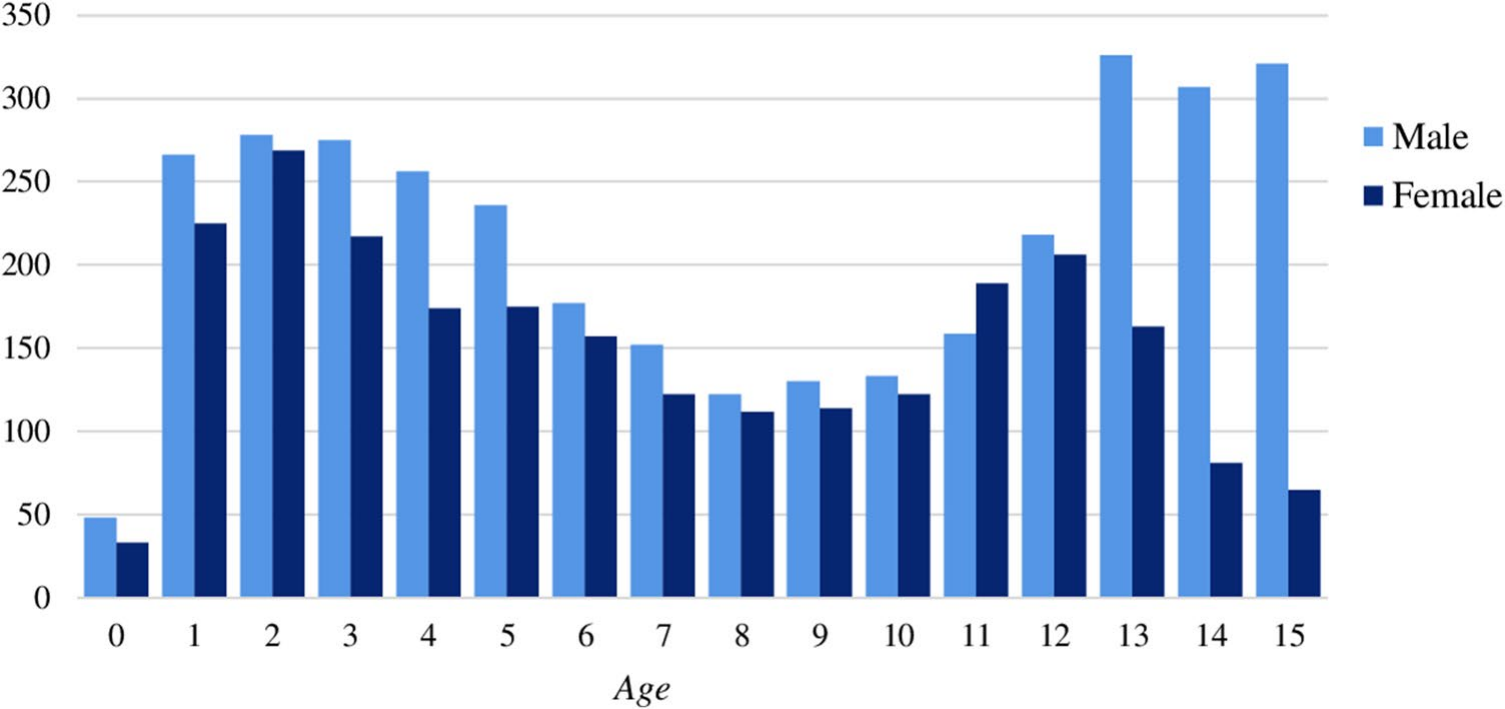
Age divided by sex distribution

5828 pediatric tibial fractures in 5719 patients between 0 and 15 years were registered between 2015 and 2019, including refractures (*n* = 109; 2%). Median age was 7 years (IQR: 3,12) and 58% of all fractures occurred in boys (*n* = 3404). Girls were statistically significantly younger at fracture than boys, median age in girls 6 years (IQR: 3,11), in boys 8 years (IQR: 3,13). The age and sex distribution shows two incidence peaks around 2 and 13 years. A larger proportion of boys were seen in the oldest age group (74%) compared to the youngest (54%) (Fig. 1).

During the study period 6 patients died, this occurred 4 to 43 months after the injury date. We have no information in this data set on cause of death. Given the time of deaths most of them should be considered unrelated to the sustained tibia fracture.

### Fracture location

Fractures of the proximal tibia accounted for 20% of all tibial fractures, 44% were shaft fractures and 36% were distal tibial fractures. Proximal and shaft fractures were equally common in the oldest age group. Shaft fractures were statistically significant more common in younger age groups while distal tibia fractures accounted for more than half of the fractures in older age groups. (Table 1). 72% of all fractures were isolated tibia fractures. The risk for isolated tibia fractures was similar between sexes (OR: 1, 95% CI 0.9–1.1) but decreased with increasing age (OR: 0.9, 95% CI 0.90–0.93).

**Table 1:** Sex, skeletal segment, common causes and activities by different age groups

Age groups	0–3	4–6	7–9	10–12	13–15
Sex (%)					
% male sex	53.8 (*n* = 867)	56.9 (*n* = 669)	53.7 (*n* = 404)	49.7 (*n* = 510)	75.5 (*n* = 954)
Skeletal segment (*n*)					
Proximal	463	199	85	120	283
Shaft	757	796	451	298	284
Distal	391	180	216	609	696
Common causes (*n*)					
Fall					
On ice/snow	31	99	50	65	75
From play equipment	386	195	103	111	65
Stumble	372	87	68	212	269
Ice- and roller skating, snow- and skateboarding etc	84	437	280	196	164
Traffic accident					
Motorcycle	0	3	12	42	142
Bicycle	33	82	41	56	74
Other transport accident	21	19	22	32	55
Activity (*n*)					
Play, free time	917	582	347	362	269
Sports	66	253	186	306	506
Other	45	15	9	13	15
Unspecified/missing	583	325	210	346	473

### Injury mechanism

#### Cause

Falls were the most common registered cause of injury (74%) with simple falls as the most common cause of injury in all age groups. Winter sport activities (ice skating, skiing, etc.) were the most common reasons for falling. Fall from play equipment and stumbles were most common causes of injury for the youngest age group (Table 1). Sport activities (skiing, snowboarding etc.) became more frequent with increasing age and had a peak in age group 4–6 years. The oldest age groups were mainly injured from sports or stumbles. Dividing different fall injuries in seasons, most falls during sport activities and on ice/snow occurred during winter and spring. Stumble occurred relatively similar during all seasons and fall from play equipment occurred mostly during spring and summer. Fall accounted for 71% of distal fractures (1487/2092), 78% of shaft fractures (2005/2586) and 72% of proximal fractures (829/1150). 56% of all patients sustaining fractures because of a fall were male.

Transport accidents accounted for 11% of all injury causes. Transport accidents were more frequent in the oldest age group (Table 1) and occurred mostly during summer. Transport accidents accounted for 14% of distal fractures (293/2092), 9% of shaft fractures (225/2586) and 10% of proximal fractures (116/1150). 68% of all patients sustaining fractures because of transport accidents were male.

#### Activity

The most common activity when sustaining a fracture was play and free time (43%) followed by sports (23%). Unspecified or missing data accounted for 33.3%. Play and free time decreased with higher age, whereas sports increased with age (Table 1). Play/free time accounted for 37% of distal fractures (772/2092), 45% of shaft fractures (1152/2586) and 48% of proximal fractures (553/1150). 52% of all patients sustaining fractures during play/free time were male. Sports accounted for 24% of distal fractures (492/2092), 23% of shaft fractures (594/2586) and 20% of proximal fractures (231/1150). 66% of all patients sustaining fractures during sports were male.

#### Place

Sports facility was the most common place at time of injury (28%) followed by home (23%), school/hospital/public premises (8.4%) and street/road (1.7%). 37.7% were registered as other specified, unspecified or missing. The child’s own home was the most common place in younger ages and decreased with higher age while sports facility increased.

#### Type

Low energy trauma accounted for 74% of the fractures, 9% were high energy trauma. 17% were unknown or not applicable due to injury mechanism. High energy trauma frequency increased with age, 4% high energy trauma in the youngest age group, 11% in the middle age group and 19% in the oldest age group. 66% of all patients with high energy trauma were male. For low energy trauma, 57% of all patients were male. High energy trauma accounted for 10% of all distal fractures (202/2092), 9% of all shaft fractures (225/2586) and 8% of all proximal fractures (89/1150).

#### Open fractures

1.1% of tibial fractures were open (63/5828). 1.8% of shaft fractures (47/2586) and 0.8% of distal tibia fractures (16/2092) were open. No proximal fractures were open. Gustilo-Anderson type IIIa was the most common open fracture type (*n* = 24), meaning high energy trauma with adequate soft tissue coverage. Remaining open fractures were Gustilo-Anderson type I (*n* = 7), type II (*n* = 15), type IIIb (*n* = 2), type IIIc (*n* = 3) or unknown (*n* = 12).

### Seasonal variation

**Fig. 2:**
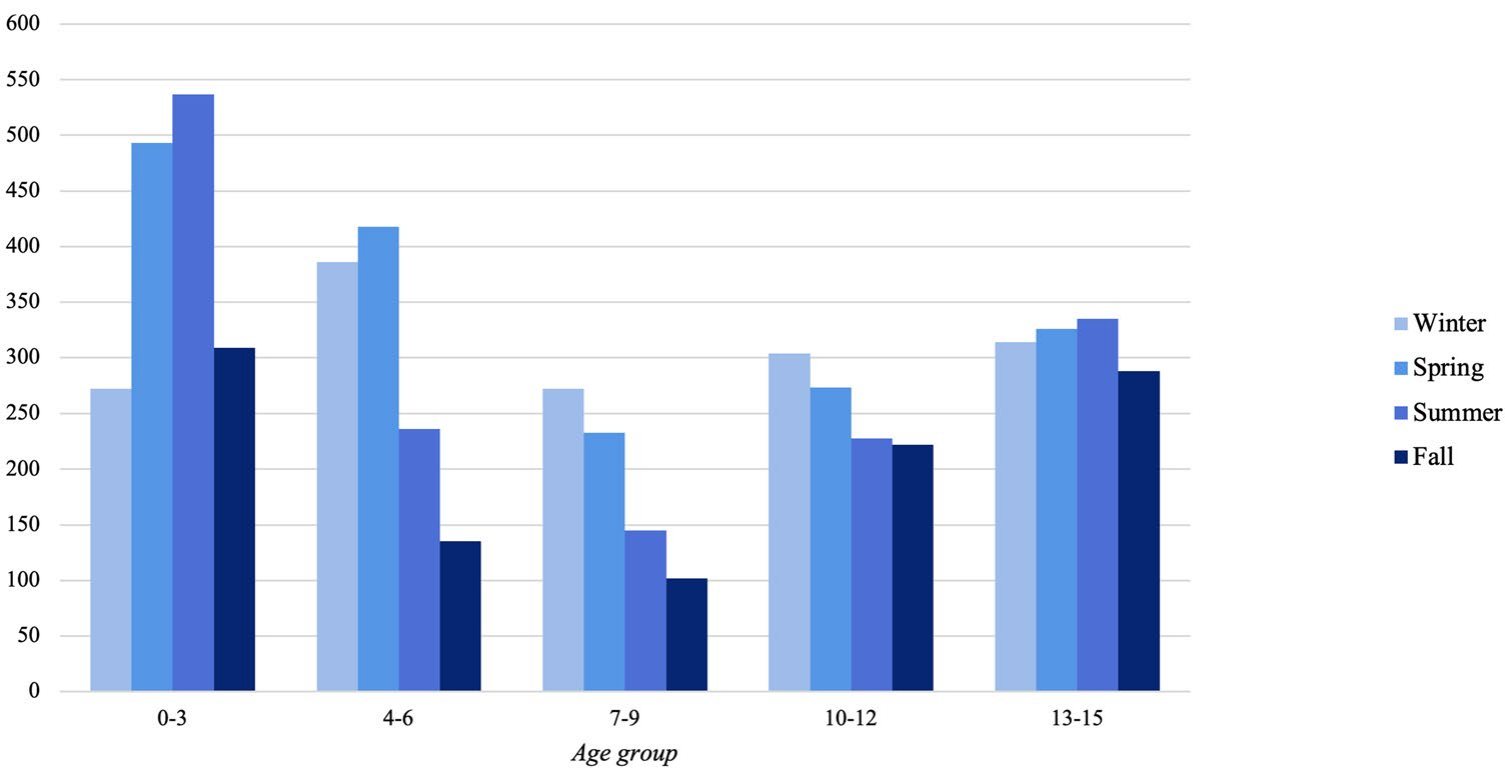
Age groups divided by seasons

30% of all fractures occurred during spring, 27% in winter, 25% in summer and 18% in fall. During winter, a statistically significant larger proportion of shaft fractures were seen. This was also the case for the risk for isolated tibia fractures (OR: 1.6, 95% CI 1.3–1.9 [ref. autumn]). Proximal tibia fractures showed no statistically significant seasonal variation. There was no seasonal variation in fracture frequency between sexes. The youngest age group had most fractures during summer and spring (Fig. 2). The oldest age group did not have a big difference in seasonal variation.

### Treatment

**Fig. 3:**
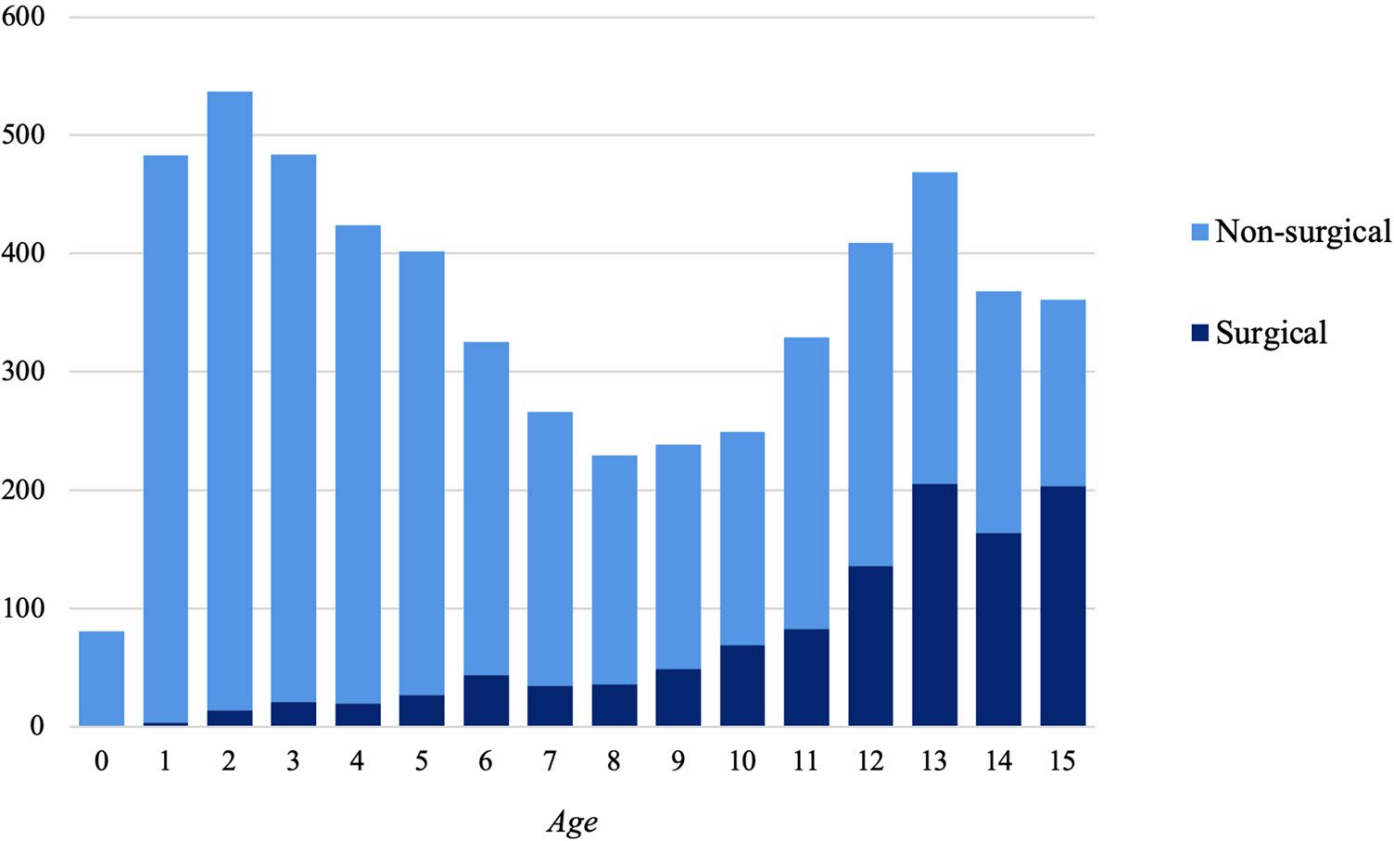
Age divided by treatment type

The vast majority of tibial fractures (78%) were treated non-surgically of these, 77% were isolated tibia fractures. 19% were treated surgically of these, 48% were isolated tibia fractures and 3% had missing information on the treatment choice. When studying treatment options, the risk for surgery increased with increasing age (OR: 1.3, 95% CI 1.27–1.33), female sex (OR: 1.5, 95% CI 1.3–1.7), and two-bone engagement (tibia and fibula) (OR: 1.68, 95% CI 1.62–1.72) (Fig. 3).

#### Treated under anesthesia

Of 1104 fractures treated under anesthesia, 288 fractures were treated with fracture reduction under anesthesia followed by casting/orthosis/other bandaging (287 closed and 1 open reduction). Of these 288 fractures, 11% were proximal, 36% shaft fractures and 53% distal tibial fractures. For the remaining fractures receiving surgical treatment, screw fixation was most common (52%) followed by K-wire/cerclage fixation (14%) and intramedullary nail (14%). Divided in fracture locations, most distal fractures were treated with screw fixation (70%) followed by K-wire (16%). Most surgically treated shaft fractures were treated with intramedullary nail (66%) followed by external fixation (17%). For proximal fractures, most surgically treated fractures were treated with screw fixation (51%) followed by K-wire (17%). Screw fixation was the most common surgical treatment type in proximal and distal fractures, while intramedullary nail was most common in shaft fractures (Table 2).

**Table 2:** Surgical treatment by fracture locations

	Proximal (%)	Shaft (%)	Distal (%)	Total
Screw fixation	87 (51)	1 (0.6)	336 (70)	424
K-wire (and/or cerclage fixation)	29 (17)	10 (6)	78 (16)	117
Intramedullary nail	0 (0)	109 (66)	4 (0.8)	113
Other fracture fixation or combined method	24 (14)	7 (4)	28 (6)	59
External fixation	3 (2)	28 (17)	11 (2)	42
Plate fixation	3 (2)	9 (6)	25 (5)	37
Fixation with bio implant	19 (11)	0 (0)	0 (0)	19
Other surgery	3 (2)	0 (0)	0 (0)	3
Extraction of implant	1 (0.6)	0 (0)	0 (0)	1
Total	169	164	482	815

### Level of treating physician

In 78% of all fractures treated under anesthesia, a consultant orthopedic surgeon was the main treating physician. One third of these orthopedic specialists had > 50% pediatric orthopedics per ordinary work week. An orthopedic resident was registered as main treating surgeon in 15% of all surgically treated fractures. Three fractures were treated by an intern physician as main surgeon.

## Discussion

Tibial fractures in Swedish children are less likely to occur in autumn but are mostly seen in winter, spring and summer. Especially isolated tibia fractures are likely to occur in winter differs from the variation of some international studies [7, 21]. Other findings in this study such as sex distribution, fracture localization, treatment choice and cause of injury are in line with previously published international studies.

The overall peak of tibial fractures during spring could be a result of two common causes overlapping: winter sports and transport accidents. These findings differ from a study on pediatric fractures by Cooper et al. from the UK stating that fractures, when studying fractures in all locations, mostly occur during summer [7]. The difference in seasonal variation might be a result of difference in activity level or type of activity between populations. For example, a high fracture rate due to skiing/winter sports in Sweden is observed during February/March, when Swedish children have school breaks. Unfortunately, Cooper et al. did not analyze different injury mechanisms which makes comparison between the results more difficult. Another possible explanation is that the UK study included fractures in general and not only tibia fractures. Different types of fractures have different injury mechanisms, possibly occurring at different times of the year, which could further explain the difference. A study by Lyons et al. comparing fracture incidence (all sites) in children from different sites in Europe concluded that the explanation for the seasonal differences likely is climatic, where the children’s activity levels during winter in Scandinavia stay relatively high due to the presence of ice and snow, whereas activity declines in Wales [16].

In the present study, we found some age variations in fracture occurrence e.g., during play and free time in younger children, whereas sports became more common with higher age. Regarding sports, previous studies have exemplified high risk sports in relation to incidence of injury. A study on adolescents in Edinburgh stated football, rugby and skiing as most common sports when sustaining a sports-related fracture [21]. This study was however on fractures in general and not only tibial fractures. Falls during skate boarding, skiing, rollerblading and skating are concerned as moderate trauma while ball sports, wrestling, judo, karate and gymnastics are categorized as low energy sport injuries, according to Landin ‘s modified trauma levels [6].

Even though non-surgical treatment was used for the majority of patients, which is in accordance with earlier conducted studies on pediatric fracture treatment [1, 9, 12, 13, 15], an increasing incidence of surgical treatment was observed with higher age. This could be explained by an increase in bone maturation with age leading to a decreased remodeling capacity and longer fracture healing. The higher frequency of surgically treated fractures in older patients could also be linked to a higher incidence of transport accidents and high energy trauma in the oldest age groups, injuries presumably in more frequent need of surgery. Female sex constituted most of the surgically treated cases for patients aged 5–12 years, which partially could be explained by a higher degree of bone maturation for girls in earlier ages. Boys had a higher rate of surgical treatment in age 13–15 years, possibly explained in part by male patients more often getting injured in transport accidents and sport activities, which could result in fractures more often in need of surgical treatment. The distribution is also in relation to injury incidence as most patients in age group 13–15 years sustaining fractures were boys.

The findings of this study regarding injury mechanisms are of use when working preventively to decrease pediatric injuries. Identifying injury patterns facilitates choosing which areas to work with in different age groups. For example, focusing on reducing risk-taking behavior in traffic for teenagers, safety work in ski slopes or safety adjustments in ski bindings, and focusing on safety precautions in playgrounds for younger children. Previously mentioned study on fracture incidence (all sites) in different European sites by Lyons et al. found lower fracture rates in the Scandinavian pediatric population [16]. This difference could, according to the authors, be explained in part by lower participation in sports such as soccer and rugby in the Scandinavian pediatric population. However, even with a relatively low incidence and low rates of complications, it is of value to minimize the risk of sustaining fractures with risk of long-term complications such as physeal growth arrest with possible leg deformity or leg length discrepancy with long follow-up time [22, 23]. Also, even though complications following pediatric fractures are uncommon, patients must manage short-term consequences such as pain, immobilization with cast or having to go through surgery.

### Strengths and limitations

This is to our knowledge the first nationwide population-based study on tibial fractures in children in Sweden based on data from SFR. The greatest strength of this study is the size of study population, with 5828 fractures included and the national design. However, as many register studies there are some sources of possible errors. At the start of the pediatric registration in 2015 36 of 54 orthopedic departments were active in the SFR. This increased to 49 at the end of 2019 (91% coverage). This stepwise introduction of the SFR makes it impossible to report on incidence rates with this study design. Completeness of the registrations is controlled annually by comparison with the National Patient Register. Completeness of pediatric tibia fractures was 42% in 2019 which is probably an underestimation as described by Bergdahl et al. [24] and have continued to increase to 58% in 2021 due to the stepwise introduction of this national quality register. Also, some orthopedic departments register only surgically treated tibia fractures due to logistic reasons.

The fractures do not always have all variables registered, resulting in groups of “missing” or “unspecified.” Registrations are time-consuming and unspecified fracture causes or locations are probably partly due to human factors, for example if the treating physician forgot to ask or register when meeting the patient. Comparing the distribution of missing data we found no sex-differences, but more missing data were found in younger ages up to 8 years compared to 9 years and older. A slightly bigger proportion of missing data was seen for shaft fractures compared to proximal and distal fractures. A few more distal fractures had missing data on place of injury.

Another limitation of this study is the risk of reporting bias. One hospital accounted for 17% of all fractures in the register, which is a large proportion of fractures for 1 out of 20 units. The county with the most fractures registered coincides with the clinic from which the Swedish fracture register originates [25]. The SFR is a relatively new register, and the differences seen in reporting frequency between counties could signify that increased awareness of the register in all counties would result in a more well-documented data nationwide. Differences are also due to new units starting to report to the SFR during the study period. This risk of reporting bias may also lead to an overreporting of surgically treated cases. Many non-surgically treated fractures are treated by other physicians than orthopedic surgeons, possibly leading to a lower registration rate compared to the surgically treated fractures. The proportions of the two treatment groups are however in line with previous studies, which could imply that this concern is of lower significance.

With the risk of reporting bias in mind, further research is encouraged focused on how the possible reporting bias affect the results. An observational study comparing statistics in the National Patient Register in Sweden with the SFR could for example display how the unregistered fractures are distributed and if they contribute to a different result.

## Conclusion

This study provides epidemiological data on tibial fractures in children in Sweden. Characteristics and injury mechanisms vary depending on age. The younger child is often injured at home during play and free time because of a fall from play equipment or stumble, resulting in a shaft fracture. Adolescents are injured while participating in sports or because of a transport accident, resulting in a distal fracture. Seasonal variation differs from international studies, being more common in spring and winter in Sweden. Overall, male sex and fall at home or at sports facilities are predominant and fractures are mostly treated non-surgically. High-energy trauma and open fractures are rare. There are different areas where incidence peaks are observed, where preventive work could be of use. For example, safety work in ski slopes and playgrounds, and improving protective equipment in sports.

## Data Availability

The dataset analyzed in this study is not freely available since the study was approved on the grounds of ensuring the confidentiality of patient data and owing to national regulations. Similar data can be obtained from the register authorities after approved ethical application.
